# The digital scribe

**DOI:** 10.1038/s41746-018-0066-9

**Published:** 2018-10-16

**Authors:** Enrico Coiera, Baki Kocaballi, John Halamka, Liliana Laranjo

**Affiliations:** 1 Australian Institute of Health Innovation, Macquarie University, Level 6 75 Talavera Rd, Sydney, NSW 2109 Australia; 2 Beth Israel Deaconess Medical Center, Boston, USA

**Keywords:** Health services, Translational research

## Abstract

Current generation electronic health records suffer a number of problems that make them inefficient and associated with poor clinical satisfaction. Digital scribes or intelligent documentation support systems, take advantage of advances in speech recognition, natural language processing and artificial intelligence, to automate the clinical documentation task currently conducted by humans. Whilst in their infancy, digital scribes are likely to evolve through three broad stages. *Human led* systems task clinicians with creating documentation, but provide tools to make the task simpler and more effective, for example with dictation support, semantic checking and templates. *Mixed-initiative systems* are delegated part of the documentation task, converting the conversations in a clinical encounter into summaries suitable for the electronic record. *Computer-led systems* are delegated full control of documentation and only request human interaction when exceptions are encountered. Intelligent clinical environments permit such augmented clinical encounters to occur in a fully digitised space where the environment becomes the computer. Data from clinical instruments can be automatically transmitted, interpreted using AI and entered directly into the record. Digital scribes raise many issues for clinical practice, including new patient safety risks. Automation bias may see clinicians automatically accept scribe documents without checking. The electronic record also shifts from a human created summary of events to potentially a full audio, video and sensor record of the clinical encounter. Digital scribes promisingly offer a gateway into the clinical workflow for more advanced support for diagnostic, prognostic and therapeutic tasks.

Nothing appears to cause more frustration for many clinicians than the electronic health record (EHR). The EHR has been associated with decreased clinician satisfaction, increased documentation times, reduced quality and length of interaction with patients, new classes of patient safety risk, and substantial investment costs for providers.^1–3^ Current generation EHRs, which rely on clinicians to either type or dictate notes, typically are also not on their own sufficient to improve patient outcomes, but do improve the quality of clinical documentation.^4^

This is because modern EHRs are, in form and function, a digital translation of paper-based records. EHR design is predicated on the primacy of the documentation task, and rarely pays heed to the other tasks within the clinical encounter. Record keeping, rather than being a by-product of the patient encounter, has become its primary orchestrator. Our conversation with the patient has been replaced by computation of the record.^5^ There is thus a strong case that the EHR, whilst necessary for effective care, is in dire need of reinvention.^6^

Clinical scribes, more common in North America, were introduced to reduce the burden of electronic documentation on clinicians. Scribes are trained to work with clinicians, translating information in clinical encounters into meaningful and accurate records, and allow clinicians to better focus on the clinical aspects of the consultation. Having scribes on the team can improve revenue and patient/provider satisfaction, and may also improve patient throughput.^7–9^ Some argue scribes are an impediment to the evolution of EHR technology, because they reduce the pressure on innovation,^10^ but this EHR ‘workaround’ also liberates clinicians to focus more on patients.^11^ More likely, human scribes are a role model for a new generation of documentation technology – the digital scribe.

Digital scribes employ advances in speech recognition (SR), natural language processing, and AI to provide clinicians with tools to automatically document elements of the spoken clinical encounter.^12–14^ We thus appear to be on the cusp of a major change in the way electronic documentation is undertaken. However, while AI may allow us to reconceive the clinical documentation task, the risks and benefits of this rapidly emerging new class of clinical system remains largely unstudied. There is little consensus on the core features that should constitute a scribe, and little exploration of how best to craft the human–computer interaction between clinician, computer and patient. In this paper, we develop a framework to understand the different capabilities that might be found in digital scribes, and describe the likely trajectory of system evolution that scribes will follow. The risks and benefits of this potentially transformational technology class are also explored.

## Intelligent documentation support systems will take over many routine clinical documentation tasks

**Fig. 1:**
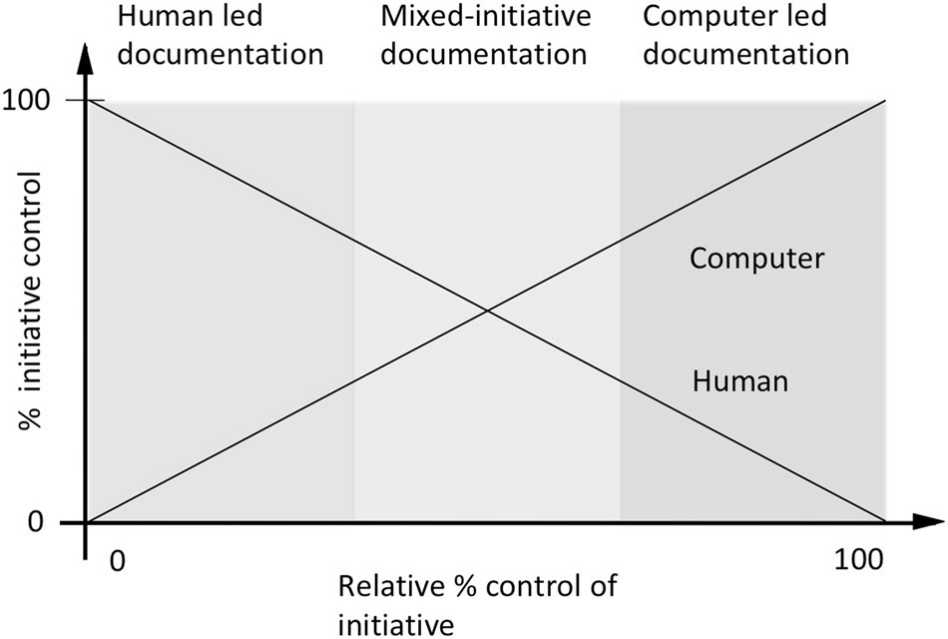
Clinical documentation systems can be strictly passive, where humans are tasked with data entry, through increasingly sophisticated mixed-imitative systems that take over more of the documentation task, to essentially autonomous or ‘autopilot’ systems that take charge of documentation. Clinical decision support functions can be embedded in the documentation process with increasing sophistication, to the point that documentation disappears as a foreground task and clinical processes become primary

Many documentation tasks can in principle be automated, either using present day technologies, or emerging AI methods. Digital scribes are *intelligent documentation support systems*. This emerging class of technology, still loosely defined, is designed to support humans in documentation tasks (Fig. 1). Such systems are well known in other sectors, such as the software industry, where they have been used in some form for over 30 years to assist with software documentation, but remain in their infancy in healthcare.^14^ There is a continuum of possible levels of such automation, commencing with humans carrying out all critical functions, through to tasks being entirely delegated to technology.^15^ In the middle, humans and computer work in tandem, each carrying out the tasks best suited to their capabilities.^16^

As the technologies needed to support rich human–computer interaction mature, such as SR and summarisation, we will likely see clinical documentation support evolve through three broad stages, each characterised by increasingly autonomous functional capabilities (Table 1):

**Table 1:** Functional characteristics of digital scribe systems

	Exemplar functions	Description
Human led documentation	Standard templates, paragraphs and macros	Common documentation tasks or content are predefined and called when needed
	Speech recognition and transcription	Speech recognition (SR) technologies create verbatim transcripts of human speech
	Automated proofing	Combining natural language processing and access to biomedical vocabularies, text can be checked for potential semantic errors.
	Simple digital assistants	SR can be used to issue voice commands to navigate the EHR;
Mixed-initative documentation	Conversational interaction model	Documentation context, stage or content can be indicated by human interaction with the documentation system using predefined gestures, commands or conversational structures.
	Computer generated summary of encounter	Extractive and abstractive text summarisation methods convert speech and other data gathered in an encounter into a succinct summary, requiring knowledge both about record structure as well as relevant biomedical knowledge.
	EHR triggered decision aids	Clinical decision support systems can be invoked at any point from data gathering to treatment decision, suggesting additional questions or observations, and alternate diagnoses, tests and treatments.
Computer-led documentation	AI will be expert in the form and content of clinical encounters, encounter records, and utilise rich models of the knowledge base underpinning specific clinical domains.	“Autopilot” systems that automatically document clinical encounters, and only prompt humans in exceptional circumstances.
Intelligent clinical environment	Ambient listening	High fidelity location aware SR coupled with speaker identification allows speech driven interaction anywhere within an environment.
	Multiple sources of sensor acquired data	Fusion and interpretation of signals from motion detection, video, clinical instrumentation, and user commands allow recording of physical examination and measurements.
	Advanced digital assistants	Detected events and machine-recognised context trigger situationally appropriate decision aids and record content, e.g. dynamic critiquing and refinement of the clinical encounter.

The processes of supporting clinical documentation and supporting decisions can be richly supported by a variety of technologies. The opportunities to re-engineer the clinical encounter away from documentation and towards decision-making increase as digital scribe systems become more autonomous, and the clinical environment becomes digitally enabled with interaction technologies for speech and gesture recognition, sensor fusion, and artificial intelligence

### Human led documentation

In today’s state of the art systems, humans are still the ones tasked with creating clinical documentation, but are provided with tools to make the task simpler or more effective. Dictation technologies are widely used to support documentation in settings such as radiology where letters and reports are a major element of the workflow. SR technologies can create verbatim transcripts of human speech,^17^ or can be used to invoke templates and standard paragraphs to simplifying the burden of data entry. SR appears to be beneficial for transcription tasks, reducing report turn-around time, but when compared to human transcriptionists, does have a higher error rate and documents take longer to edit.^18^

SR is also now increasingly used not just for note dictation, but as the primary mechanism to interact with the EHR. SR can be used to navigate within the EHR, select items in drop down lists, and enter text in different fields of the record. Despite the wide availability of such technology, it has surprisingly had little formal evaluation. When evaluations are performed, they suggest that SR leads to higher error rates, compared to the use of keyboard and mouse (KBM), and significantly increases documentation times.^19^ This suggests that, while SR is useful for dictating long notes, it may not be an ideal mechanism for interacting with complex environments like the EHR.

This may be simply because SR to date is “bolted on” to a system primarily designed with KBM in mind. There also appear to be fundamental cognitive limits to the use of SR. Speech processing requires access to human short-term and working memory, which are also required for human problem solving.^20^ In contrast, when hand–eye coordination is used for pointing and clicking, more cognitive resources are available for problem solving and recall. This means that experienced keyboard users have a greater ability to problem solve in parallel while performing data entry, compared to those using SR.

Some EHRs now incorporate decision-support to automatically proof dictated text for obvious linguistic and clinical errors, next word auto-completion or suggest items commonly associated with information already entered, for example proposing additional investigations or diagnoses consistent with a note’s content. Assistive features can predict the likely content of notes, such as gender, age through to overall note structure, using inferred templates.^21^

Whilst in principle such prompting can be helpful, it needs to arrive at a point in the clinical process where it can be of value. Suggesting tests or diagnoses at the end of a consultation when an assessment is complete and tests have been ordered might just be too late to make a clinical difference.

### Mixed-initiative documentation systems

This emerging class of documentation support models itself more on human scribes and is delegated part of the documentation task. Human and computer each take the initiative for some parts of the record generation process, and record generation emerges out of the partnership.

Automated documentation systems in this class of digital scribe must automatically detect speech within the clinical encounter and use advanced SR to translate the discussions and data associated with an encounter into a formal record. Clinicians might interact with digital scribes using voice commands or hand gestures (much as we do with home assistant systems), or may use augmented reality technologies, such as smart glasses. Documentation context, stage or content can be signalled by human interaction with the documentation system using predefined gestures, commands or conversational structures.

Key to understanding the technical leap required to develop such systems is the distinction between present day transcription systems and still emergent summarisation technologies. Today’s speech systems are designed to detect and then literally transcribe each word that is spoken. Automated documentation systems are also tasked with recognising speech, but must then create a summary or précis of its content, suitable for documentation. By analogy, a transcriber is like a gene sequencer, literally creating a read of all the ‘words’ in a DNA sequence, without addition or deletion. A summarizer in contrast must identify only what is salient to the encounter, like sifting junk DNA from coding sequences. It must then communicate it’s meaning, just as we are ultimately interested in the functional role of a gene rather it’s constituent base pairs. How that might best happen is still the subject of research.

Text summarisation methods are traditionally broken down into *extractive* methods, which identify and extract unchanged salient phrases and sentences, and *abstractive* methods, which produce a shortened reinterpretation of text based on inference about its meaning.^22^ When a summary is generated from human speech instead of a set of documents, additional tasks emerge, such as speaker identification and SR, as well as more classic natural language processing tasks. These include mapping recognised words and phrases to a common language reference model,^23^ and the use of hybrid methods, such as rules to populate pre-defined templates, e.g. for well-defined sections of a clinical note such as medication or allergies.^14^ Deep learning methods can be used in tandem with such approaches, or on their own.^17,24^ Once a machine readable summary is created, methods for the automated generation of text from such structured representations can create a human readable version of the information.^25–27^ Whilst much effort is currently focussed on automating the summarisation process, it should not be forgotten that humans are a ready source of context cues. Many difficult problems in natural language processing may be solved by good human–computer interaction design.

### Computer led documentation

A third class, the “autopilot” digital scribe, will emerge when computers can lead in the documentation process. Human interaction would only occur to assist the machine in resolving specific ambiguities in the clinical encounter, perhaps to clarify goals and intentions, request missing details, or resolve contradictions. For highly structured and well-bounded encounters, for example routine clinic visits to monitor patients for chronic illness or post-operative recovery, the entire documentation process might be delegated to automation, and humans only invoked when exceptions to the expected process occur.

Achieving this class of documentation system will require major advances in AI, and much experience with the use of the less autonomous versions of digital scribes described earlier. Not only will autonomous documentation systems need to be expert in the form and content of clinical encounters, and the encounter record, they will need to exploit rich models of the knowledge base underpinning specific clinical domains.

## Intelligent clinical environments

Advances in human–computer interaction technologies, such as haptic control and virtual reality, as well as biomedical sensor design, are also likely to have significant impact upon digital scribe design. Rather than the focus being on engaging with a defined stand-alone ‘computer’, the environment itself can become the computer.

Sensors in the clinical workspace will capture a wide variety of data for the EHR. Cameras can record clinical interactions and store them either as a full video record, or only extract summary information, such as positions or events to preserve privacy. Data from clinical instruments such as a digital otoscope can be automatically transmitted, interpreted using AI and entered directly into the record. Clinicians and patients can interact with the intelligent environment using a combination of gestures and speech, or by touching a variety of active surfaces.

Once a clinical encounter occurs in a fully digitised space, the locus of human–machine interaction transforms. Interaction occurs wherever humans decide it must, and the machine, afforded multiple ways to sense what is happening, can become much more context-aware and adaptive.^28^ More foundationally, our conception of the nature of the patient encounter, as supported by technology, will need to be refashioned.

While some EHR manufacturers are perhaps unsurprisingly first focussing on using smart environments to detect billable events such as specific physical examinations, the potential benefits of working in such a setting are profound. Today’s clinical decision support (CDS) technologies will find a new and more useful entry point into the clinical workflow in digital environments. While CDS have been repeatedly demonstrated to have an impact on clinical outcomes,^29^ getting them used has been another matter. In an intelligent environment, CDS can be refashioned to join the human–machine conversation in a manner that should see them used far more effectively.

Diagnostic algorithms might gently and privately prompt clinicians to ask additional questions at specific moments in an encounter, or to seek additional clinical signs or order additional tests. A clinician can quickly enquire of an information retrieval system about information from the past clinical record or clinical knowledge sources, just at the moment that the information need arises. Critiquing systems, a form of AI long studied in academia,^30^ can examine a clinician’s proposed treatment plans and suggest modifications. Suggestions might be based on guidelines, or on an analysis of data from similar past patients,^31^ bringing personalised medicine directly into the clinical encounter.

## Risks and challenges of digital scribes

Digital scribes raise a number of important issues for clinical practice. As with all information technology, digital scribes bring new patient safety risks. As we have seen, SR works well for note dictation, but is less effective when used with modern EHRs, introducing errors with the potential for patient harm.^19^ Digital scribes provide a way to reimagine the use of SR, not as a means to navigate and populate an EHR, but to support the natural information needs that arise in a clinical encounter. To achieve this, speech technology needs to improve further, with its design and use subject to rigorous safety principles.

Developing the AI systems underpinning digital scribes will require access to clinical data sets for machine learning. In keeping with other clinical applications, there are ethical and privacy challenges in re-using data for algorithm development.^32^ Amongst these is the creation of privacy preserving anonymised data sets. Anonymising speech records of encounters is especially challenging. Data that identifies the patient, their clinician, other providers, and carers or relatives are likely to pepper clinical talk and be difficult to mask.

Automation bias, where clinicians incorrectly follow instructions from technology, are an important new cause of clinical error,^33^ and there are similar risks that clinicians will automatically accept scribe suggestions or completed documents without checking. There will also be a temptation to create more detailed records than currently produced. The time cost and difficulty of reviewing long documents could erode some of the efficiencies gained through the technology.

As the nature of the record changes, there will be significant medico-legal ramifications. The “ground truth” of the record will shift from human produced text to a machine generated summary, potentially associated with a full audio, video, and sensor record of the clinical encounter.^34^ The summary record will thus become a layer of interpretation on top of this ground truth, potentially open to revision. There will be pressure to retain raw encounter data to allow future clinicians to reinterpret them in the light of later events, or for quality improvement and population health research. Counter arguments that raw data should be deleted and only the interpretation retained can be made from a defensive medico-legal viewpoint.

Implementation science is making clear that similar systems implemented in different settings are likely to generate different outcomes due to variations in local context such as resources, practices, and patient case-mix.^35^ Clinical encounters are highly variable, not just between settings and specialties, but across nations. We should thus expect that linguistic and cultural differences are likely to make it difficult to directly translate digital scribe designs across nations, and considerable effort may be needed to adapt and extend digital scribes.

With the advent of Europe’s General Data Protection Regulation (GDPR), other issues also come into play. For instance, Article 22 states that “the data subject shall have the right not to be subject to a decision based solely on automated processing”. A digital scribe by definition would thus always require a clinician to sign off on the final document, and patients might need to explicitly consent to have their record created in such a way.

Finally, scribe technologies will no doubt transform the nature of the clinical encounter and the relationship between clinician and patient. How that plays out will depend much on the way interactions are designed with scribes. Replacing typing directly into the EHR with intrusive commands to a speech interface, or time spent checking and signing off records, might gain us little.

## Conclusion

The digital scribe has the potential to enhance clinical encounters and re-emphasise patient care over documentation. Digital scribes offer a gateway into the clinical workflow for advanced decision support for diagnostic, prognostic and therapeutic tasks. Their arrival is thus likely to be transformational for clinical practice. There remain however significant challenges ahead, extending from the technical to the professional. Critically, such changes will require the active engagement of the clinical community, focussing on maintaining the quality and safety of the clinical encounter and its record. It will also require clinicians and patients to be leaders in re-imagining how they wish to work together, assisted by technology.
