# Author Correction: The digital scribe

**DOI:** 10.1038/s41746-018-0069-6

**Published:** 2018-10-30

**Authors:** Enrico Coiera, Baki Kocaballi, John Halamka, Liliana Laranjo

**Affiliations:** 1 Australian Institute of Health Innovation, Macquarie University, Level 6 75 Talavera Rd, Sydney, NSW 2109 Australia; 2 Beth Israel Deaconess Medical Center, Boston, USA

**Correction to**: *npj Digital Medicine* 10.1038/s41746-018-0066-9, Article Published online 16 Oct 2018

The original version of the published Article contained an error in the spelling of the third Author’s name. “John Halamaka” has been changed to “John Halamka”. This has been corrected in the HTML and PDF version of the Article.

